# Electrocatalytic Reactions for Converting CO_2_ to Value-Added Products: Recent Progress and Emerging Trends

**DOI:** 10.3390/ijms24129952

**Published:** 2023-06-09

**Authors:** Zohreh Masoumi, Meysam Tayebi, Mahdi Tayebi, S. Ahmad Masoumi Lari, Nethmi Sewwandi, Bongkuk Seo, Choong-Sun Lim, Hyeon-Gook Kim, Daeseung Kyung

**Affiliations:** 1 Department of Civil and Environment Engineering, University of Ulsan, Daehakro 93, Namgu, Ulsan 44610, Republic of Korea; zohrehmasoumi17@gmail.com (Z.M.); tonethmisewwandi@gmail.com (N.S.); 2 Center for Specialty Chemicals, Division of Specialty and Bio-Based Chemicals Technology, Korea Research Institute of Chemical Technology (KRICT), Jonggaro 45, Ulsan 44412, Republic of Korea; mtayebi2900@gmail.com (M.T.); bksea@krict.re.kr (B.S.); chsunlim@krict.re.kr (C.-S.L.); 3 Department of Energy Engineering and Physics, Amirkabir University of Technology, Tehran 15875-4413, Iran; mehditayebi2784@gmail.com; 4 Department of Biology, York University, Farquharson Life Sciences Building, Ottawa Rd, Toronto, ON M3J 1P3, Canada; sinasmasoumi2004@gmail.com

**Keywords:** electrochemical reaction, CO_2_ conversion, reduction reaction, microbial electrosynthesis

## Abstract

Carbon dioxide (CO_2_) emissions are an important environmental issue that causes greenhouse and climate change effects on the earth. Nowadays, CO_2_ has various conversion methods to be a potential carbon resource, such as photocatalytic, electrocatalytic, and photo-electrocatalytic. CO_2_ conversion into value-added products has many advantages, including facile control of the reaction rate by adjusting the applied voltage and minimal environmental pollution. The development of efficient electrocatalysts and improving their viability with appropriate reactor designs is essential for the commercialization of this environmentally friendly method. In addition, microbial electrosynthesis which utilizes an electroactive bio-film electrode as a catalyst can be considered as another option to reduce CO_2_. This review highlights the methods which can contribute to the increase in efficiency of carbon dioxide reduction (CO_2_R) processes through electrode structure with the introduction of various electrolytes such as ionic liquid, sulfate, and bicarbonate electrolytes, with the control of pH and with the control of the operating pressure and temperature of the electrolyzer. It also presents the research status, a fundamental understanding of carbon dioxide reduction reaction (CO_2_RR) mechanisms, the development of electrochemical CO_2_R technologies, and challenges and opportunities for future research.

## 1. Introduction

Since the industrial revolution of the 19th century, fossil fuels such as petroleum, natural gas, and coal have been used as the main source of energy to power economies and civilizations [1]. There is a need to reduce CO_2_ emissions because the burning of these fossil fuels has resulted in excessive CO_2_ emissions into the atmosphere, which have had significant negative effects on the environment and pose an immediate threat to human societies [2,3,4]. The swift transformation of the need of energy and chemical industries from fossil fuels to renewable energy resources, for example, solar and wind, can be identified as one of the solutions to achieve the closed-looped configurations on the carbon footprint [5,6,7].

Nonetheless, several artificial solutions to limit or reduce CO_2_ emissions have been created, such as technological innovation to increase coal burning efficiency in boilers (reducing coal consumption) and carbon capture and sequestration (CCS) [8,9,10] though CCS is a costly and an energy-consuming technology. In fact, dangerous CO_2_ leakage is a major concern that inhibits the commercialized large-scale deployment of CCS. As a result, fixation of CO_2_ remains a significant concern on a global scale [11,12,13]. 

Hence, currently, the best strategy is to use atmospheric CO_2_ as a renewable feedstock to create a few chemical products with added value, such as light olefins, urea, formic acid, methanol, syngas, and (poly)carbonate [14]. A technique such as this will reduce the atmospheric CO_2_ levels while producing fuels and industrial chemicals, reducing the reliance on traditional fossil fuels [15,16]. Therefore, several CO_2_ reduction strategies, such as photochemical, electrochemical, thermochemical, and biochemical procedures, have been developed and extensively researched [17,18].

Among these technologies, lowering CO_2_ emissions using renewable power is especially tempting due to its enormous potential, simple reaction units, controlled selectivity, and modest efficiency for practical industrial applications [19]. Furthermore, it is possible to think of electrocatalytic carbon dioxide reduction (ECR) as a useful method for storing the renewable energy discussed above in chemical forms [20,21,22,23,24]. ECR paired with renewable energy techniques as electricity sources are widely employed in the energy sectors and chemicals, and it may offer a promising route to create considerable amounts of chemicals and carbon-neutral fuels [25,26]. Electrochemical CO_2_ conversion offers various benefits over other methods: (i) using renewable energy sources such as solar, wind, geothermal, and tidal; (ii) the mechanism is simpler and precise in terms of administering as it only requires the monitoring of reaction temperatures and the potential of electrodes; (iii) having scalable, compact and highly efficient on demand transmutation systems; (iv) hydrocarbons can be formed from water, carbon dioxide, and renewable electricity [27,28].

The main question is how to build a high-performance CO_2_ conversion system that has all the desired qualities at the same time [29]. The main component of a high-performance CO_2_ conversion system is a system that has higher operational current density and produces better faradaic and energy efficiency for CO_2_R [30,31]. Many research efforts in ECR have been directed to the search for better electrocatalyst materials, because appropriate electrocatalysts have a better active site that ideally leads to the synthesis of desirable products at high rates and low overpotentials [32,33,34,35].

Metals, metal oxides, two-dimensional materials, and functional microorganisms have all been investigated as CO_2_ reduction electrocatalyst materials. Metals’ catalytic durability, selectivity, and activity could be improved by controlling their crystal faceting, morphology, and size [36]. There are activities for electrocatalytic CO_2_ reduction in metal oxides such as Co_3_O_4_ [37,38], CuO [39,40], ZnO [41,42], and TiO_2_ [43,44]. Contrary to pure metal catalysts, most CO_2_ reduction process intermediates are expected to bind via their oxygen atoms and those of metal oxides. This criterion implies that metal oxides have higher oxygenate selectivity than pure metal catalysts [45,46]. 

Two-dimensional (2D) materials with nanosheets can exhibit unique features and great performance in catalytic processes when used as catalysts. Two-dimensional electrocatalysts decrease the energy barrier for CO_2_ activation, improve electrical conductivity, and have a high surface-active site density, which makes them promising for highly efficient CO_2_ conversion [47,48]. Because, as compared to ordinary bulk materials, they have a significantly higher percentage of bare surface atoms and higher specific surface areas, they might provide an abundance of active sites, enhancing catalytic processes [49,50]. It should be noted that highly exposed surface atoms might escape and create defect structures, resulting in lower coordination numbers of surface atoms, which are attractive locations for reactant or intermediate adsorption. Similarly, nanosheet edge atoms with low coordination numbers can display unique catalytic characteristics. As a result, 2D structures can boost reactant chemisorption and improve catalytic efficiency [51].

Bio-catalysis, which incorporates microbes and enzymes, has received a great deal of interest because the value-added products can be produced under mild circumstances with remarkable selectivity and without any undesirable byproducts [52,53]. Given previous research in bio-inspired molecular structure design, expanded and dynamic connections through the materials, biological, and chemical science domains will synergistically promote catalyst development [49,50]. Microbial electrosynthesis (MES) utilizes self-replicating bacteria as a catalyst at room temperature and pressure, which enables a more economical and ecologically benign process than traditional chemical catalyst-based conversion. To metabolize CO_2_, bacteria in MES exchange electrons directly or indirectly using electron shuttle molecules [54]. To recycle anthropogenic CO_2_, electroactive microorganisms are employed in MES as a biocatalyst on suitable electrode materials [55].

Therefore, the current review covers a brief background of the efforts and strategies undertaken in the scientific community to improve electrocatalytic reactions for converting carbon dioxide into value-added products in terms of electrocatalyst materials and their morphology, electrolyte, temperature, pressure, and applied voltages. Furthermore, the newest accomplishments of microbial electrosynthesis for CO_2_ conversion are discussed. In addition, some intriguing reaction mechanisms linked to electrocatalysts are described and discussed. Finally, in this fascinating area of research, potential future difficulties and outlooks of electrocatalytic CO_2_ conversion into value-added products are proposed.

## 2. Concepts of Electrochemical CO_2_ Reduction Reaction

### Concepts of Electrochemical CO_2_ Reduction Reaction

The electrochemical conversion of CO_2_, a linear stable molecule with a powerful C–O bond (750 kJ mol^−1^), is challenging. Multi-electron/proton transfer processes, a large variety of possible reaction intermediates, and an ECR in an aqueous electrolyte are all part of the extremely complicated process of ECR [56,57].

Electrochemical reduction has been researched in aqueous solutions with various metal cathodes, as well as in several organic solvents. Although the successfully documented six-electron and eight-electron conversions to methanol and methane exist, the commonly discussed reduction products are carbon monoxide, acetic acid, and formic acid [58,59,60]. The main ECR products’ half electrochemical thermodynamic reactions are shown in Table 1, ethanol (CH_3_CH_2_OH), ethylene (C_2_H_4_), formic acid (HCOOH), methanol (CH_3_OH), methane (CH_4_), carbon monoxide (CO), and acetate (CH_3_COOH), with reporting of their standard redox potentials at acid and base electrolytes [61,62].

**Scheme 1:**
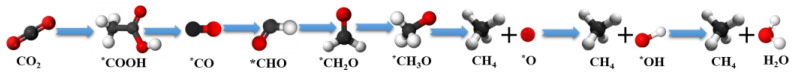
Pathway for the electrochemical conversion of methane from CO_2_.

In an ECR process, CO_2_ molecules adsorb on the catalyst surface and interact with the atoms there to produce *CO_2_, which is then followed by many progressive transfers of electrons and/or protons toward different end products. For instance, methane is thought to originate via the pathways given below (Scheme 1) [63]: 

A multistep reaction process, electrochemical CO_2_ reduction typically involves a different number of electron reaction pathways. The reaction frequently happens at the electrolyte–electrode interface for heterogeneous catalysts used in CO_2_ reduction, where the electrode is typically a solid electrocatalyst and the electrolyte is typically an aqueous solution saturated with CO_2_ through bubbling.

Water is transformed to oxygen and CO_2_ is reduced to the CO_2_ anion radical at the anode in a single-electron ECR (CO_2_^−^). The first step of converting CO_2_ to reduced carbon species is difficult because the reaction rate is very slow. The single-electron CO_2_ reduction to CO_2_^−^ with a pH of 7 exhibits an unfavorable and energetic reaction, with a thermodynamic potential of roughly −1.90 V vs. SHE. Furthermore, the formation of the CO_2_ intermediate is essential to the formation of the 2e^−^ reduction products and the initial process can be considered the rate-limiting step [64].

**Figure 1:**
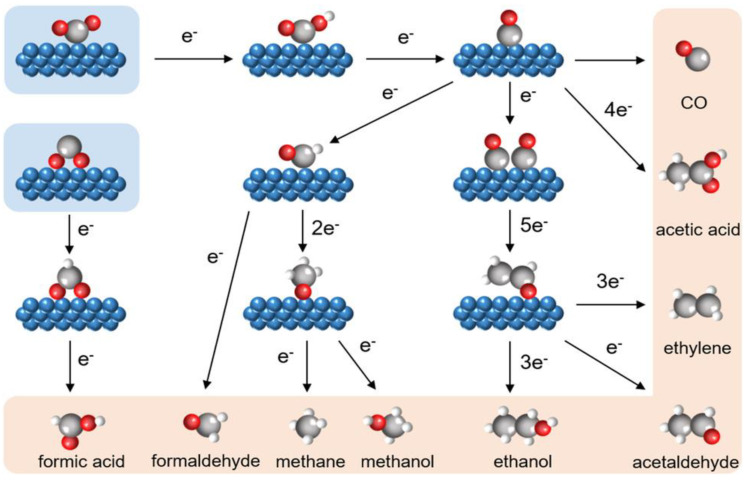
An overview of CO_2_RR’s reaction pathways leading to various products [64].

Several electron/proton transfer processes are involved in the electrochemical CO_2_RR, and CO_2_ can be reduced into a collection of gaseous and liquid products by diverse pathways, including hydrocarbons (CH_4_ and C_2_H_4_), alcohols (CH_3_OH and C_2_H_5_OH), carbon monoxide (CO), and formic acid (HCOOH) [65]. This depends on the electrolytic conditions and the electrocatalysts used (e.g., applied potential, electrolyte, etc.) [28,37,66]. Without a catalyst, it is challenging to complete the first stage of CO_2_ activation, which produces the intermediate CO_2_^−^ radical. However, with the aid of an electrocatalyst, the CO_2_^−^ radical can be stabilized via a chemical link created between CO_2_ and the electrocatalyst, leading to less negative redox potential. Moreover, proton-coupled electron transfer is advantageous at the likely range of 0.20 to 0.60 V vs. SHE. The end products are influenced by the electrocatalyst and electrolyte selections as well as the quantities of electrons and protons transferred [51]. Therefore, the activation routes of some typical products in CO_2_RR are briefly shown in Figure 1 [64].

Molecule reactants may react with various CO_2_RR intermediates at any phase since CO_2_RR contains several reaction steps and intermediates, which greatly broadens the range of possible products. Consequently, potential products can be selectively derived through the adjustment of the adsorption and desorption capability of electrocatalysts to distinct reaction intermediates from coupled CO_2_RR [64]. 

**Figure 2:**
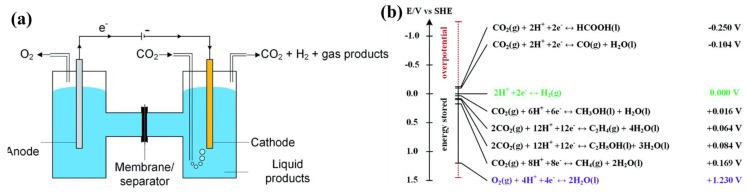
(**a**) Electrochemical CO_2_ reduction in an H-cell reactor [67]. (**b**) At 25 C and 1 atm, standard equilibrium potentials for the half-cell hydrogen evolution and other CO_2_ reduction reactions [36].

A laboratory electrochemical H-cell consists of oxygen evolution reaction (OER) happening at the surface of an anode that generates electrons (e^−^) and protons (H^+^) or consumes hydroxyl ions (OH^−^); a cathode in order to reduce CO_2_ to produces such as HCOOH/HCOO^−^ or CO, and make OH^−^; an electrolyte with the intention of transporting CO_2_ to the active cathode sites and conduct ions; a membrane that allows ion exchange to take apart the anode and cathode; and a bias with suitable value to move electrons from anode to cathode (Figure 2a). A few crucial steps in a CO_2_R process are involved in such a system, including (1) movement of products into liquid phases or bulk gases from the cathode/electrolyte interface, (2) product desorption from the electrode, (3) transfer of electrons from the cathode to intermediates, (4) adsorption of CO_2_ into adsorbed intermediates such as *CHO, *CO, and *COOH, (5) the surface of the cathode absorbing CO_2_, (6) transport of dissolved CO_2_ to the cathode/electrolyte interface from the bulk electrolyte, and (7) CO_2_ mass transfer to the bulk electrolyte from the gas phase [67].

One of the most critical problems of the electrochemical CO_2_R technologies to function at large-scale is to obtain a great CO_2_ selectivity to desired value-added products to reduce product separation costs and complexity. High selectivity is difficult to achieve due to, as shown in Figure 2b, the majority of CO_2_R reactions’ standard potentials (Eo) and the hydrogen evolution reaction (HER) all being within a limited variety (−0.250 V to 0.169 V vs. standard hydrogen electrode) (SHE) [36].

## 3. Product Selectivity Parameters

The applied potential, pressure, temperature, type of electrolyte (pH, concentration, and composition), and type of electrocatalyst (crystallographic structure, chemical state, composition, and morphology) are all variables that affect selectivity, FE, and ECR performance.

In addition, the selectivity of catalysts for various products varies. The type and quantity of electrolytes also affect the catalyst’s activity and selectivity. While C_2_ products (such as ethanol, ethylene, and acetic acid) have primarily been observed using copper-based catalysts, C_1_ products (such as CO, methane, methanol, and formic acid) can develop in a variety of materials [68,69]. Therefore, we primarily concentrated on several electrolytes and catalyst architectures for the conversion of CO_2_.

### 3.1. Electrocatalyst Materials and Their Morphology

#### 3.1.1. Metals

A CO_2_ radical anion is produced by electrochemical reduction with an inert metal or carbon electrode. This radical anion can be dimerized to form oxalate or disproportionated to form CO and carbonate. Active metals, on the other hand, may direct CO_2_ reduction to hydrogenated products via active sites on their surface at a significantly reduced voltage applied. The metal in these systems performs a dual purpose, supplying electrons while also stabilizing the reduced pieces. 

In the 1980s, Hori performed the key study that first established CO_2_RR. In this work, he demonstrated the ability of several pure metal catalysts to reduce CO_2_, which paved the way for extensive CO_2_R research [70]. He demonstrated that pure metal catalysts could be classified into four groups: (1) transition metals that primarily produce CO, such as Au, Ag, Zn, and Pd; (2) main group metals that produce formate (HCOO^−^), such as Pb, In, and Sn; (3) metals with negligible activity toward CO2RR, such as Ni, Fe, Pt, and Ti; and (4) Cu, which can produce hydrocarbons and multicarbon products [71].

**Figure 3:**
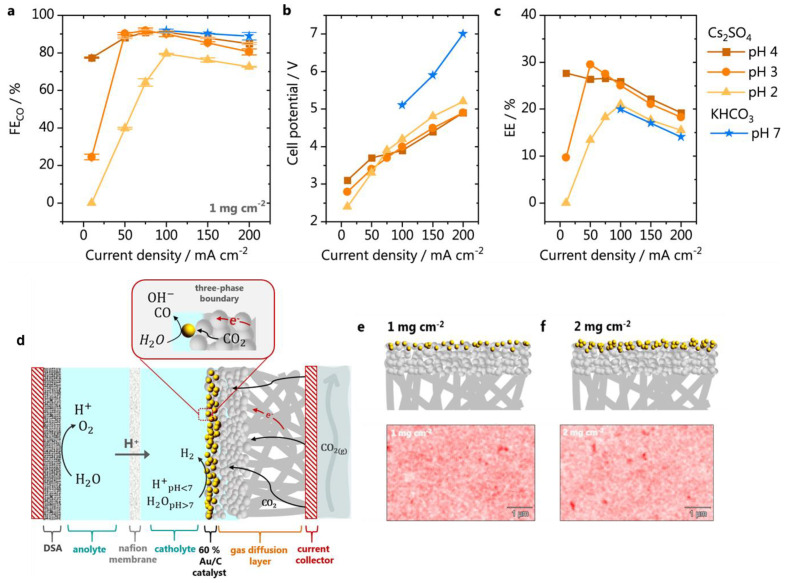
(**a**) FE for CO; (**b**) cell potential; (**c**) energy efficiency for electrolysis of CO_2_. (**d**) The system of gold gas-diffusion electrodes is shown schematically. (**e**,**f**) Gold GDEs are shown schematically with loadings of 1 mg cm^−2^ and 2 mg cm^−2^, respectively, and an EDX elemental map of the gold GDEs [72].

Monteiro et al. [72] conducted CO_2_R in varying current densities in sulfate electrolytes (100–200 mA cm^−2^) using a 10 cm^2^ gold gas-diffusion electrode. According to their findings, moderately acidic media can support high CO selectivity (90%) at 100–200 mA cm^−2^, provided insufficiently hydrated cations (Cs^+^, K^+^) are available in the electrolyte. They discovered that in 1 M Cs_2_SO_4_ electrolyte, CO_2_R can be performed at notably lower cell potentials than in a neutral medium (1 M KHCO_3_), resulting in a decrease of up to 30% in process energy expenditures. According to recent findings in idealized small-scale DEMS measurements that proton reduction can be suppressed during CO_2_R under suitable conditions, their results show that FEs of 80–90% for CO can be obtained at a gold gas-diffusion electrode at current densities up to 200 mA/cm^2^, demonstrating the feasibility of running CO_2_ electrolysis in acidic media (Figure 3).

**Figure 4:**
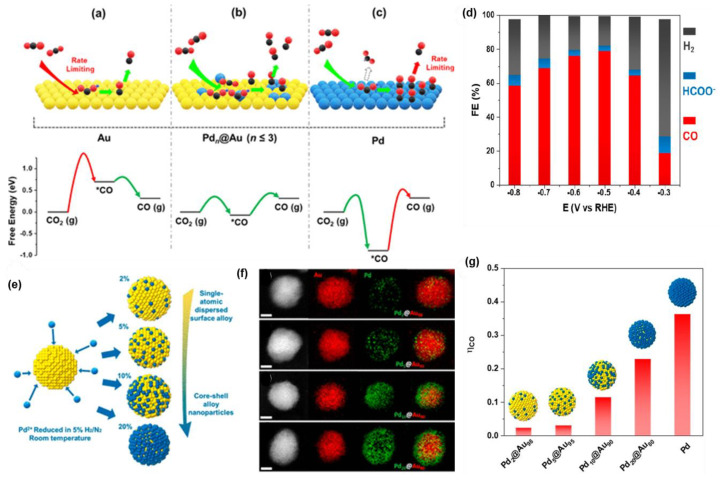
(**a**–**c**) Atomically dispersed Pd sites on the surface of Au to improve CO_2_ reduction is a representation of the concept. (**d**) For Pd5@Au95, various products’ FEs were estimated. (**e**) An illustration of the Pd@Au nanoparticles synthesis strategy with Pd dose control. (**f**) From top to bottom, STEM illustrations and element maps for Pd2@Au98, Pd5@Au95, Pd10@Au90, and Pd20@Au80. (**g**) Comparison of the Pd-based electrocatalysts’ estimated CO adsorption coefficients (η_CO_) [73].

In a new method described by Wang et al. [73] (Figure 4) the number of active sites on bimetallic catalysts and their size are engineered to increase the efficiency of CO_2_ reduction. Pd was added to Au nanoparticles in a variety of regulated dosages to create Pd@Au electrocatalysts. The nonlinear dependency of their catalytic activity for the conversion of CO_2_ to CO was attributed to the fluctuation of *CO and *COOH adsorption energies on the Pd sites of various ensemble sizes. In contrast, FE_HCOO^−^_ grows from 9.8% for Pd5@Au95 to 56% for pure Pd at 0.3 V, whereas J_HCOO^−^_ rises from 0.019 to 0.059 mA/cm^2^. Bimetallic Pd-Au surfaces with discrete, atomically scattered Pd ensembles have lower energy barriers for CO_2_ activation than pure Au and are also less poisoned by strongly binding *CO intermediates than pure Pd, with Pd dimers striking a balance of these two rate-limiting variables.

#### 3.1.2. Metal Oxides

Metal oxides display an extremely wide variety of capabilities due to their various bonding, structures, and compositions. Further, defects in metal oxides give them a range of functions, and the capacity to chemically adjust the distribution, population, and type of defects in the bulk and on the surface of metal oxides provides attraction in many applications [74]. Employing heterostructures using metal and metal oxide is an efficient means of generating new chances for improved catalysis. Strong metal/oxide interactions have been frequently used to enhance the kinetics of chemical catalysis in the gas phase; however, analogous notions for electrocatalysis in the liquid phase have received far less attention [75].

**Figure 5:**
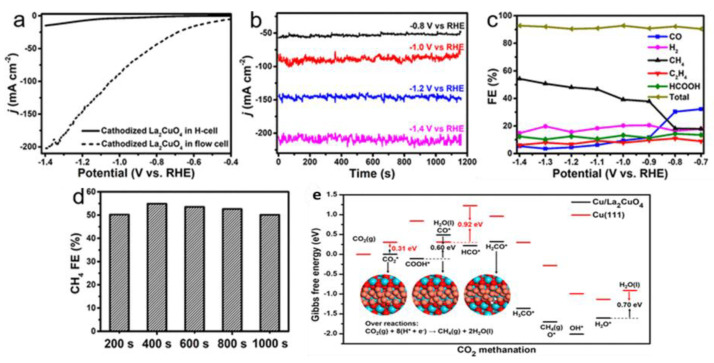
(**a**) LSV curves with a 10 mV/s scan rate in a flow cell and an H-cell. (**b**) Electrolysis in steps at various biases. (**c**) Produces electrolysis at various bias. (**d**) CH_4_ electrolysis of the catharized La_2_CuO_4_ at −1.4 V_RHE_. (**e**) Identified energy diagram for the CO_2_ methanation at the interface of Cu/La_2_CuO_4_ and surface of Cu (111) [76].

Chen et al. [76] describe the electrochemical creation of CH_4_ from CO_2_ conversion. Theoretical studies and experimental results suggest that the Cu species are decreased to metallic Cu on the surface of perovskite and partially exsolved from the perovskite, and the resulting Cu/La_2_CuO_4_ heterostructure may be the cause for the efficient CO_2_ methanation procedure. Methane was produced over this perovskite catalyst at −1.4 VRHE (Figure 5), With a current density of 117 mA/cm^2^ and a FE of 56.3%. This research demonstrates an efficient perovskite electrocatalyst for ambient electrochemical CO_2_ methanation and may provide a significant understanding of the structural evolution and surface reconstruction of electrocatalytic materials during electrochemical reactions in energy-relevant technologies.

**Figure 6:**
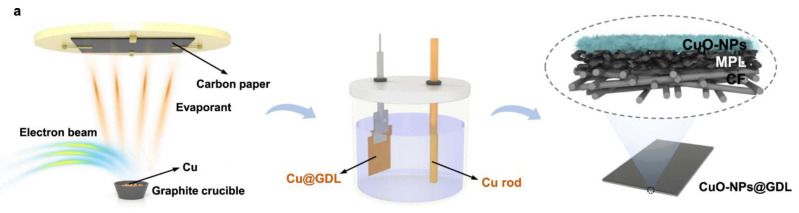

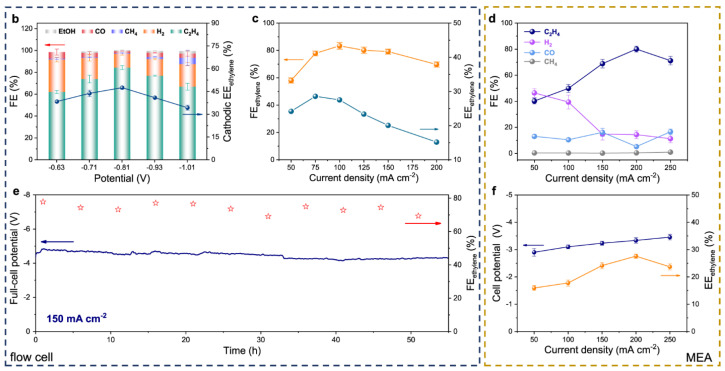
(**a**) Schematic of CuO-NPs@GDL preparation. (**b**) The flow cell’s DVL-Cu@GDL catalyst’s FEs and cathodic EE_C2H4_. (**c**) The DVL-Cu@GDL catalyst’s full-cell potential and cathodic EE_C2H4_ in the flow cell. (**d**) The DVL-Cu@GDL catalyst’s gas product FEs in a MEA electrolyzer. (**e**) Stability test of the DVL-Cu catalyst in the flow cell at a constant current density of 150 mA/cm^2^. (**f**) The DVL-Cu@GDL catalyst’s cell potential and EE_C2H4_ in a MEA electrolyzer [77].

For the large-scale production of Cu catalysts which are oxide-derived with stable Cu/Cu_2_O surfaces for highly active CO_2_RR to C_2_H_4_ with high FE and sustained stability, Liu et al. [77] (Figure 6) have developed an anodic oxidation approach. The stable Cu/Cu_2_O interfaces and high degree of oxidation of the vertically stacked Cu nanoplates on the Cu foil during the CO_2_RR preclude the clustering of nanostructures. With the help of these properties, the DVL-Cu catalyst obtains high FE_C2H4_ and EE_C2H4_ values of 84.5 and 1.7%, 28.9 and 1.3%, and 27.6 and 0.8%, respectively, in the flow cell and 200 mA/cm^2^ in the MEA electrolyzer. In fact, the DVL-Cu catalyst keeps the flow-electrolysis cell’s performance constant for about 55 h. According to density functional theory (DFT) calculations, the energy barrier for C-C coupling is dramatically lowered because Cu^+^ species increase the *OCCOH intermediate’s ability for adsorption. The DVL-Cu catalyst’s remarkable selectivity, long-lasting stability, and ease of manufacture indicate its potential for use in achieving the industrial conversion of CO_2_ to C_2_H_4_.

**Figure 7:**
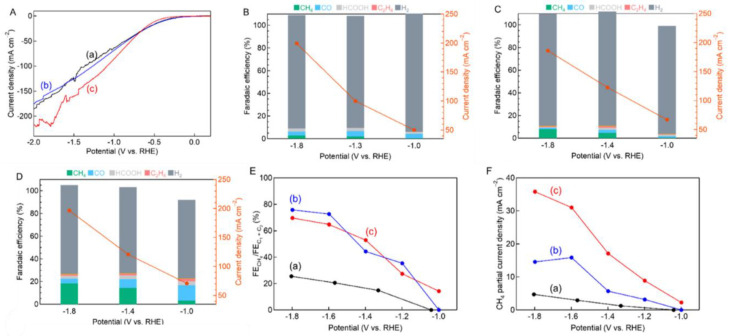
(**A**) LSV curves are shown for an electrode using TiO_2_, Cu-TiO_2_, and Cu-TiO_2_-H catalysts under CO_2_ flow. (**B**) FEs over TiO_2_, (**C**) Cu-TiO_2_, and (**D**) Cu-TiO_2_-H catalysts at various potentials for various products. (**E**) FECH_4_/FEC_1_+C_2_ comparison on catalysts made of (a) TiO_2_, (b) Cu-TiO_2_, and (c) Cu-TiO_2_-H. (**F**) Comparison of the CH_4_ partial current densities of the catalysts TiO_2_, Cu-TiO_2_, and Cu-TiO_2_-H [78].

Anzai et al. [78] (Figure 7) successfully synthesized Cu-TiO_2_ composite catalysts with well-dispersed Cu clusters or nanoparticles using a one-pot solvothermal technique after which the CO_2_ is reduced electrochemically through thermal treatment. CuO_x_ clusters were disseminated on the TiO_2_ surface in Cu-TiO_2_ samples obtained by air calcination of the precursor, while Cu NPs were produced in Cu-TiO_2_-H samples obtained by subjecting the precursor to hydrogen. Cu-TiO_2_-H was discovered to have a good selectivity for CH_4_ in electrochemical CO_2_R. At a CH_4_ partial current density of 36 mA/cm^2^ at −1.8 V_RHE_, faradaic efficiency for CH_4_ synthesis reached 18%. Moreover, 70% of FE_CH4_/FE_C1+C2_ was obtained at −1.8 V_RHE_. They believe that the homogeneity of the Cu NPs produced on TiO_2_ is one of the critical criteria for maximizing CH_4_ selectivity in the electrochemical CO_2_R.

#### 3.1.3. Two-Dimensional Materials

The most promising method for achieving a carbon-neutral cycle is CO_2_ conversion into hydrocarbon fuels, which could be accomplished with the help of advances in electrocatalysis science. The possibility for two-dimensional materials to operate as highly efficient electrocatalytic CO_2_ reduction catalysts has recently been recognized [79]. As an illustration, transition-metal dichalcogenides (TMDCs) interacting with ionic liquid (IL) electrolytes can approach a CO_2_ reduction system that benefits from materials with overlying of the d-band partial density of states with the Fermi energy, low work function, and an electrolyte “solvent” that effectively transports CO_2_ to the active site [80].

**Figure 8:**
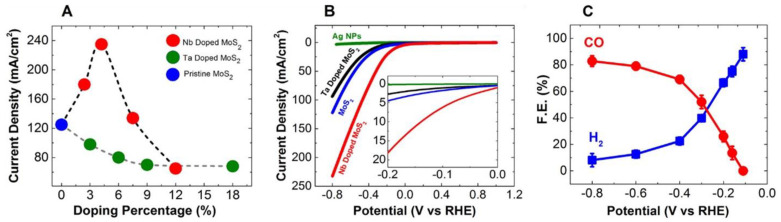
(**A**) Current density against dopant percentages of Nb and Ta. (**B**) Cyclic voltammetry curves in CO_2_ for Ag nanoparticles, VA-MoS_2_, VA-Mo_0.97_Ta_0.03_S_2_, and VA-Mo_0.95_Nb_0.05_S_2_. (**C**) CO and H_2_ faradaic efficiency (FE%) for VA-Mo_0.95_Nb_0.05_S_2_ at various applied potentials [81].

Abbasi et al. [81] (Figure 8) produced VA-Mo_1−x_M_x_S_2_ structures (M = Nb and Ta) and investigated their efficiency as electrocatalysts in the CO_2_ reduction procedure. The maximum catalytic performance was determined to be Va-Mo_0.95_Nb_0.05_S_2_ with a CO formation TOF of this structure, which was shown to be dual magnitudes greater than a Ag nanoparticle catalyst over the whole spectrum of overpotentials. The best results were obtained when the dopants in the MoS_2_ structure were embedded in the catalysts’ atomic structure, implying that this could provide a viable path to enhance the edge atoms of the catalytic performance by altering their electronic characteristics. The influence of Nb in the Mo_1−x_M_x_S_2_ structure was investigated using DFT computations. The DFT results for Ta-doped MoS_2_ also indicate that Ta doping in the second Mo row of MoS_2_ may result in an unfavorable reaction pathway, i.e., the production of COOH^*^ becomes endergonic. Although pure TaS_2_ appears to have appropriate reaction pathways, its greater work function (5.5 eV vs. 5.0 eV for MoS_2_) might be a disadvantage for its electron-transfer property. Thus, unlike Nb-doped MoS_2_, the DFT simulations revealed that Ta-doped MoS_2_ is unlikely to have a satisfactory “trade-off” effect between the reaction energetics and the work function.

**Figure 9:**
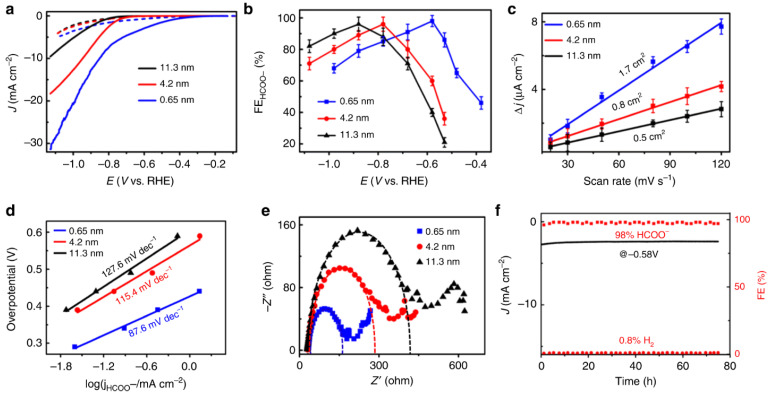
(**a**) LSV curve with pH corrected. (**b**) FE at each applied bias. (**c**) Electrochemical active surface area estimation. (**d**) Tafel graphs for different BiNSs thicknesses. (**e**) Nyquist curves for various layers of BiNS. (**f**) Bismuthene nanosheets (0.65 nm) at −0.58 V potential and associated FEs for HCOO^−^ and H_2_ have long-term stability [82].

Yang et al. [82] describe that Bismuth nanosheets (BiNSs) are produced via a scalable, facile wet chemical method, and its increased electrocatalytic CO_2_RR performance toward HCOO-synthesis is demonstrated. Bi-single-atom layers were produced for the first time due to their high atom density, outstanding electrical conductivity, the high surface density of more intrinsically active sites, and improved structural stability. With a high FE of 99%, durability (>75 h), and a low onset overpotential of 90 mV, these layers may selectively catalyze the electroreduction of CO_2_ to HCOO^−^ exclusively (Figure 9). Their theoretical research suggests that the thickset BiNSs, which expose the (011) facet, firmly bind reaction intermediates, possibly poisoning them.

#### 3.1.4. Functional Microorganisms 

Microbial electrosynthesis (MES) uses electrographic microorganisms as biocatalysts to generate compounds from CO_2_. MES can reduce CO_2_ using an electroactive bio-film electrode as a catalyst [83,84,85]. Microbial adherence is affected by the range of electrode geometries and material qualities, which impacts biofilm formation and electron exchange [86]. Microbial fuel cells provide many advantages over biomass energy production, including high energy conversion efficiency, room-temperature operation, no requirement for gas treatment, and low energy input [85].

**Figure 10:**
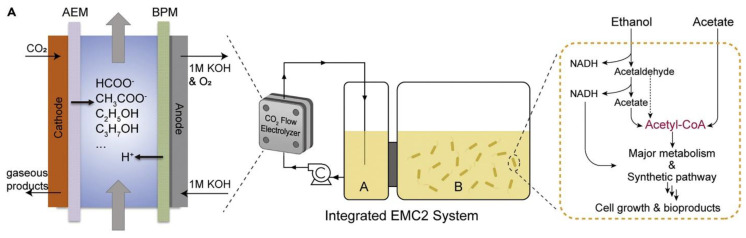

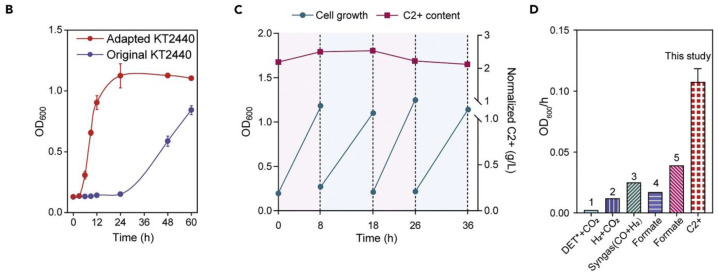
(**A**) Schematic illustration of the electrochemical–biological integrated EMC2 system. (**B**) Comparison of P. putida KT2440 WT cell growth before and after adaptation. (**C**) Profiles of C2 concentration and cell development in an integrated system. (**D**) Evaluation of the EMC2’s cell growth rate in comparison to that of other electro-microbial systems [86].

Catalytic CO_2_ reduction and bioconversion could significantly increase the amount of carbon captured and used while reducing climate change. Current technologies are unfortunately constrained by inefficient electron and mass transfers, poor metabolic kinetics, and a paucity of molecular building blocks. Zhang et al. [86] (Figure 10) overcome these challenges by using electrocatalysis, a chemical–biological (chem-bio) interface, and systematic microbial design to enable efficient electro-microbial conversion with C2 (EMC2). Faster mass transfer, simpler entry into primary metabolism, reduced toxicity, increased energy and electron transport, and superior molecular building blocks for many bacteria are all advantages of soluble C2 intermediates. The EMC2 system’s multi-tier chem–bio interface architecture increased microbial biomass productivity by six and eight times in contrast to the C1 intermediate and hydrogen-driven pathways, respectively.

**Figure 11:**
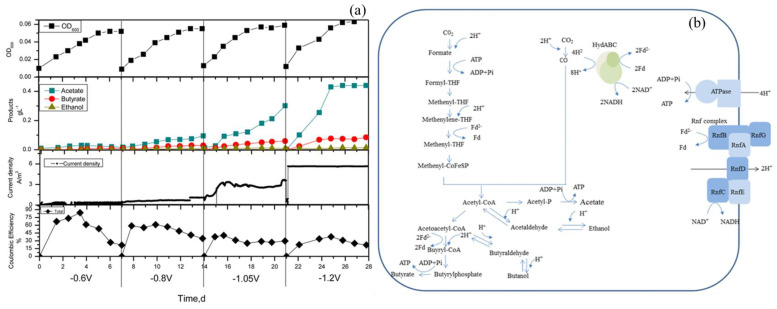
(**a**) *C. scatologenes* ATCC 25775 microbial electrosynthesis tests for OD_600_; product process; current density; and coulombic efficiency at four various potentials from top to bottom. (**b**) Based on genome sequencing, the Wood–Ljungdahl pathway of *C. scatologenes* ATCC 25775 [87].

*C. scatologenes* is an acetogenic bacterium that can fix CO_2_ via the Wood–Ljungdahl route and operate as a biocatalyst in MES systems, as described by Liu et al. [87] (Figure 11). At a potential of −0.6 V, the cathodic chamber produced the highest amounts of acetic acid and butyric acid, 0.03 and 0.01 g/L, respectively. The maximum total coulombic efficiency was approximately 84%. The LES system adopted H_2_ when the cathodic potential decreased and ethanol was found in the product spectrum. Nonetheless, due to the reduced H_2_ evolution rate, the MES system’s H_2_ usage rate fell to 37.8%. Overall, the synthesis of butyric acid, a C-4 molecule, increases the possibility for MES deployment greatly. Based on genome sequencing, direct electron transfer (DET) in the MES system is thought to be aided by hydrogenase and ATPase. Furthermore, *C. scatologenes*, like *C. ljungdahlii* and *C. aceticum*, should theoretically be capable of accepting electrons straight from an electrode.

**Figure 12:**
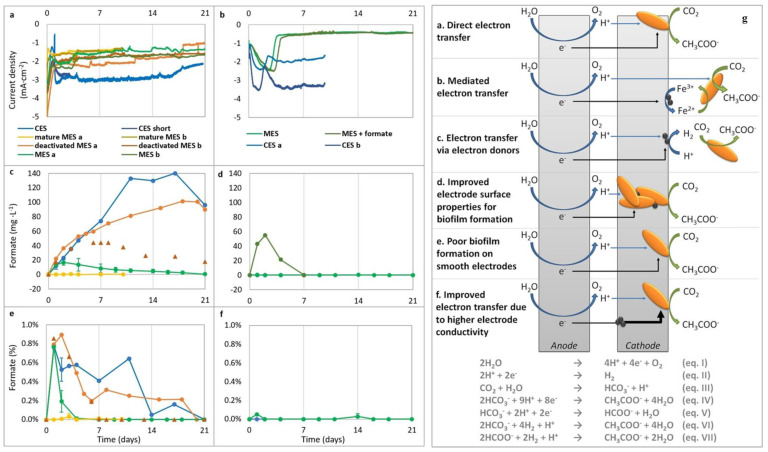
Electrode electrochemical performance throughout several chronoamperometry studies. (**a**,**b**) Current density; (**c**,**d**) dissolved formate concentration; and (**e**,**f**) predicted electron recovery with time. (**g**) The connection between a metal catalyst (for example, iron, shown as grey nanoparticles) and an MES culture is depicted schematically (illustrated as orange cells) [88].

Chatzipanagiotou et al. [88] (Figure 12) demonstrated that metabolic cooperation between copper electrocatalysts and MES biocatalysts, with formate as a metabolic intermediary, is possible. Up to 140 mg L^−1^ of formate was created entirely by copper oxide, indicating a co-catalytic (i.e., metabolic) interaction, whereas formate was also clearly formed by copper and eaten by bacteria generating acetate. The two catalysts had syntrophic effects, with CuOx electrodes resulting in a threefold increase in current density and acetate generation. Although biofilm coverage on the electrode’s surface was not explored, it was shown that the presence of microorganisms in the electrolyte had no effect on copper’s long-term catalytic activity for CO_2_ reduction to formate. Methods for improving the performance of co-catalytic ideas based on copper catalysts are reviewed, such as modifying the electrode shape and electrolyte composition. 

Here, the summary of electrocatalytic CO_2_ conversion in various electrode structures with their optimal condition is highlighted in Table 2. Table 2 shows the copper and copper oxide, copper-oxide-derived, copper-carbon catalysts, and doped copper catalysts. The dominant usage of copper can be explained by its ability to produce multicarbon products during the reduction of CO_2_. The use of Cu electrodes in CO_2_ reduction experiments allows for the formation of a broad variety of products. Thereby, it becomes visible that the selective formation of multicarbon alcohols still possesses a challenge. Ethylene is generally preferred to ethanol formation in copper-based electrodes. As can also be seen from Table 2, the most frequently used electrolytes are KHCO_3_ and KOH. One key parameter with a strong influence on catalyst/electrode performance is the electrolyte. Compared to KHCO_3_, a higher selectivity for carbonaceous products using KOH was shown. High local pH values, which can be favored by an electrolyte with low buffer capacity, have been shown to improve the product distribution toward higher hydrocarbons. Even when comparing 1 M KOH with 1 M or 0.1 M KHCO_3_, clear differences can already be seen. Although the same current densities can be achieved in principle with both electrolytes, the same current densities can be reached with 1 M KOH at considerably lower voltages; because the CO_2_RR activity is significantly higher there in a catholyte with higher basicity, less energy is therefore required for the CO_2_RR. In addition, the use of 1 M KOH also shifts the selectivity toward carbonaceous products.

## 4. Summary and Perspectives

In the last decade, significant progress in CO_2_RR has been made by analyzing reaction mechanisms and creating electrocatalysts, electrolytes, and electrolyzes. In this work, we reviewed recent reports on the building of improved and efficient electrocatalytic reactions for the CO_2_ conversion to value-added commodities using engineering approaches.

To improve electrocatalytic reactions for converting CO_2_ to value-added products, we found that there is still a dearth of knowledge, which adds to the remaining challenges. CO_2_RR based on modified metal, metal oxide, and two-dimensional materials may be viable electrocatalyst materials for converting CO_2_ to commercially valuable compounds. Sustainable energy production using electrochemical CO_2_ conversion with low environmental impact offers an excellent opportunity to reduce fossil fuel consumption.

The development of new catalyst materials and composites with computational modeling and mechanistic studies should be the focus of future research to create stable and highly effective electrocatalysts. (1) Now, a major issue for the CO_2_RR is salt precipitation brought on by carbonate formation on the cathode side, which lowers the catalyst surface’s active area. (2) There are issues about the stability and scalability of CO_2_RR research because new-structured devices can increase CO_2_ conversion efficiency and support stability. (3) The CO_2_RR aims to treat CO_2_ emissions from fossil fuel combustion in real-world scenarios. The requirement for flue gas CO_2_ capture is eliminated by directly substituting feed gas with flue gas from the combustion of fossil fuels. This process necessitates a large amount of energy as well as additional purification units.

## Figures and Tables

**Table 1:** Standard redox potentials (V_RHE_) for ECR generation processes in acid and base.

Produces	Acidic Electrolyte		Alkaline Electrolyte	
	Chemical Reactions	Potential	Chemical Reactions	Potential
Ethanol	2CO_2_ + 12H^+^ + 12e^−^ → CH_3_CH_2_OH + 3H_2_O	0.084	2CO_2_ + 9H_2_O + 12e^−^ → CH_3_CH_2_OH + 12OH^−^	−0.744
Ethylene	2CO_2_ + 12H^+^ + 12e^−^ → C_2_H_4_ + 4H_2_O	0.085	2CO_2_ + 8H_2_O + 12e^−^ → C_2_H_4_ + 12OH^−^	−0.743
Formic acid	CO_2_ + 2H^+^ + 2e^−^ → HCOOH	−0.171	CO_2_ + H_2_O + 2e− → HCOO^−^ + OH^−^	−0.639
Methanol	CO_2_ + 6H^+^ + 6e^−^ → CH_3_OH + H_2_O	0.016	CO_2_ + 5H_2_O + 6e− → CH_3_OH + 6OH^−^	−0.812
Methane	CO_2_ + 8H^+^ + 8e^−^ → CH_4_ + 2H_2_O	0.169	CO_2_ + 6H_2_O + 8e− → CH_4_ + 8OH^−^	−0.659
Carbon monoxide	CO_2_ + 2H^+^ + 2e^−^ → CO + H_2_O	−0.104	CO_2_ + H_2_O + 2e− → CO + 2OH^−^	−0.932
Acetic acid	2CO_2_ + 8H^+^ + 8e^−^ → CH_3_COOH + 2H_2_O	0.098	2CO_2_ + 5H_2_O + 8e^−^ → CH_3_COO^−^ + 7OH^−^	−0.653

**Table 2:** Summary of the main types of CO_2_RR electrocatalysts and their optimal condition.

Electrocatalyst	Catalyst Loading	Electrolyte	Main Product	Total Current Density	FE (%)	Reference
PdSn/C	0.5 mg cm^−2^	0.5 M KHCO_3_	HCOOH	2 mA cm^−2^ at −0.43 V vs. RHE	99	2017/[89]
Pd-B/C	100 μg cm^−2^	0.5 M KHCO_3_	Formate	10 mA cm^−2^ at −0.5 V vs. RHE	70	2018/[90]
MOF-AuPd	1.0 mg cm^−2^	0.5 M KHCO_3_	HCOOH	7 mA cm^−2^ at −0.25 V vs. RHE	99	2021/[91]
Pd_80_Ag_20_/C	-	0.5 M NaHCO_3_ /0.5 M NaClO_4_	HCOO^−^	11 mA cm^−2^ at −0.18 V vs. RHE	97.8	2019/[92]
Gold gas-diffusion electrode	1 mg cm^−2^	1 M KHCO_3_	CO	100 mA cm^−2^ at −0.18 V vs. RHE	90	2021/[72]
Pd_5_@Au_95_	10 μg cm^−2^	0.1 M KHCO_3_	CO	1.6 mA cm^−2^ at −0.5 V vs. RHE	80	2019/[73]
Cu/La_2_CuO_4_	0.12 mg cm^−2^	1 M KOH	CH_4_	12 mA cm^−2^ at −1.4 V vs. RHE	56.3	2020/[76]
Cu/Cu_2_O	-	1.0 M NaOH	C_2_H_4_	200 mA cm^−2^ at −0.81 V vs. RHE	84.5	2022/[77]
Cu-TiO_2_	-	1 M KOH	CH_4_	117 mA cm^−2^ at −1.8 V vs. RHE	70	2022/[78]
β-Bi_2_O_3_	1 mg cm^−2^	1 M KOH	Formate	288 mA cm^−2^ at −0.61 V vs. RHE	98	2019/[29]
VaMo_0.95_ Nb_0.05_S_2_	1 mg cm^−2^	EMIM-BF_4_	CO	237 mA cm^−2^ at −0.8 V vs. RHE	83	2017/[81]
BiNSs	0.39 mg cm^2^	0.5 M KHCO_3_	HCOO^−^	2.5 mA cm^−2^ at −0.58 V vs. RHE	98	2020/[82]

## Data Availability

Not applicable.
